# Commentary: The effects of postponing BCG vaccination on the risk of BCG-related complications among patients with severe combined immunodeficiency disease in Saudi Arabia

**DOI:** 10.3389/fimmu.2026.1832253

**Published:** 2026-05-28

**Authors:** Amr Ahmed, Hiba K.H. Abdelseed

**Affiliations:** 1 Public Health Department, Riyadh First Health Cluster, Ministry of Health, Riyadh, Saudi Arabia; 2 Tuberculosis Control Program, Public Health Department, General Directorate, Ministry of Health, Riyadh, Saudi Arabia

**Keywords:** 6 months, BCG - Bacille Calmette-Guérin vaccine, infants, meningitis, postpone, SCID - Severe Combined Immunodeficiency, tuberculosis, vaccination

## Introduction

We read with interest the article by Aldhaheri et al. which is published at Volume 16 - 2025 | https://doi.org/10.3389/fimmu.2025.1596963 (1) reporting reduced BCG-related complications in SCID patients following the Saudi Ministry of Health’s 2019 decision to postpone BCG vaccination from birth to 6 months. The study demonstrated a significant decline in BCGitis from 46.1% to 2.6% (p < 0.001) at King Faisal Specialist Hospital and Research Center (KFSH-RC). While this finding is clinically relevant for SCID management, we identify several critical methodological limitations that constrain the study’s applicability to national vaccination policy.

## Absence of population-level impact assessment

The study’s most consequential limitation is its exclusive focus on SCID patients (n=178) without assessing the impact on the general neonatal population. With a Saudi SCID incidence of 1:2,906 live births (2), for every SCID infant protected, approximately 2,905 immunocompetent neonates remain unprotected for 180 days. Saudi Arabia’s TB incidence is 8.4 per 100,000 (95% UI: 7.6–9.3) in 2023, reflecting a 63.5% reduction since 2000 (3), and BCG provides 70–80% protection against severe childhood TB forms including meningitis (4). The authors relegate this gap to a single sentence under Limitations, yet this is not a minor omission it fundamentally undermines the conclusion that the revised policy “provides a model for optimizing immunization strategies.” A policy evaluation that measures benefits without quantifying costs is inherently incomplete.

## Failure to compare with the 28-day model

The study presents a binary comparison BCG at birth versus 6 months without discussing the internationally adopted alternative. In September 2021, the UK Joint Committee on Vaccination and Immunization (JCVI) moved BCG to 28 days of life, linked to TREC-based neonatal SCID screening (5, 6). The JCVI modelled that this short delay would not significantly increase TB cases while protecting SCID infants (7). Slovenia (2025) and the Netherlands employ similar approaches, conditioning BCG on screening results rather than imposing prolonged delays (8). By omitting this comparison, the study obscures a demonstrably safer middle path that achieves both objectives: SCID safety and timely TB protection.

## Uncontrolled confounding and study design limitations

Era 2 (August 2019–January 2023) overlaps almost entirely with the COVID-19 pandemic, which disrupted diagnostic services, referral pathways, and surveillance systems globally (9). No multivariate analysis or sensitivity analysis was conducted to disentangle the policy effect from pandemic-related confounders. Furthermore, the single-center tertiary referral design (KFSH-RC) introduces selection bias patients represent the most severe end of the SCID spectrum and do not reflect the general population experience. The two BCGitis cases in Era 2 were both diagnosed with SCID late (at 10 and 22 months), suggesting that delayed SCID diagnosis not vaccination timing per se was the primary risk factor, which is an argument for TREC screening rather than prolonged BCG delay.

## Vaccine coverage and missed vaccinations

The study does not report BCG coverage rates before and after the policy change. The study does not discuss the defaulter rate following the schedule change. The current Saudi NIP policy precludes BCG administration after 12 months (10), meaning children who miss the 6-month window are permanently unprotected. The WHO emphasizes that BCG should be given at birth or the earliest opportunity to avoid missed opportunities (11), and that birth-dose delivery achieves the highest coverage because of the captive audience in maternity facilities.

## Non-specific effects of neonatal BCG

Neonatal BCG confers heterologous protective effects (“trained immunity”) mediated by epigenetic reprogramming of innate immune cells, reducing all-cause neonatal mortality (12, 13). A 2025 multicentre RCT in India demonstrated significant mortality reductions in low-birth-weight neonates receiving BCG at birth (14). These non-specific benefits, accruing from the first days of life, are entirely forfeited during a 6-month delay and were not considered by the authors.

The current binary choice delay BCG and leave immunocompetent neonates unprotected from TB, or vaccinate at birth and risk BCGosis in undetected SCID patients represents an incomplete framing. The scientific evidence reviewed here identifies two biologically coherent alternative paths that may be pursued in parallel.

## Failure to quantify the population-level cost of 180-day unprotected period

Perhaps the most critical omission in Aldhaheri et al.’s analysis is the absence of any comparative assessment between the benefit conferred upon the SCID population estimated at 1 in 2,906 live births in Saudi Arabia (2) and the documented harm inflicted upon the remaining 2,905 immunocompetent neonates who were left unprotected against tuberculosis for 180 days. A scientifically rigorous policy evaluation must weigh both sides of this equation. The study reports a reduction in BCGitis from 46.1% to 2.6% among SCID patients, yet presents no data on TB incidence, disseminated TB, tuberculous meningitis, ICU admissions, or TB-related mortality among the unvaccinated birth cohorts following the 2019 policy change. This is not a minor gap; it is a fundamental methodological failure that undermines the study’s central conclusion.

This concern is amplified by the sociodemographic reality of Saudi Arabia’s population. A substantial proportion of births occur among expatriate workers, undocumented residents, and low-income families who face significant structural barriers to healthcare access, including language barriers, fear of legal consequences, financial constraints, and limited awareness of vaccination schedules. Unlike the captive-audience opportunity at birth where BCG can be administered universally within the maternity facility regardless of residency status a 6-month schedule shift requires a deliberate return visit that many vulnerable families will not make. Children born to undocumented or low-income families, who are paradoxically at highest risk of TB exposure due to crowded living conditions and delayed care-seeking behaviour, are thus most likely to remain permanently unvaccinated a consequence invisible in single-centre tertiary referral data but profoundly real at the population level.

“Bernatowska et al. (2022) demonstrated that the BCG Moreau substrain produced in Poland was associated with significantly fewer cases of BCGosis in patients with SCID and other inborn errors of immunity compared with Brazilian and other substrains. Notably, the Danish substrain historically used in Saudi Arabia prior to this policy change has been associated with a higher propensity for BCGosis compared with other substrains, as evidenced by data from 17 countries encompassing 349 SCID patients. This substrain-specific variability in adverse-event profiles represents an additional contextual factor that the authors did not address and that may partially account for the high BCGitis rates observed in Era 1” (15).

Two screening tests either TST or IGRA are not reliable at this age 6 months, so Delayed BCG vaccination at six months may subsequently develop devastating disseminated BCG disease (BCGosis), raising two unresolved questions of critical clinical importance. First, whether pre-existing subclinical Mycobacterium tuberculosis latent infection (LTBI) acquired during the unprotected window period acts as an immunological cofactor precipitating dissemination of the live attenuated BCG strain upon vaccination. Second, whether the tuberculin skin test (TST/PPD) the most widely available diagnostic tool provides sufficient sensitivity and specificity at six months of age to serve as a pre-vaccination safety screen.

Finally there is critical point as when given the all unvaccinated babies the BCG vaccine at 6 months of age this is considered masked procedure. And critical clinical scenario so the critical practical implication is that neither TST nor IGRA, used alone or in combination, provides a sufficiently reliable screen for LTBI in infants under six months to serve as the sole safety checkpoint before delayed BCG vaccination. The clinical scenario for postponed BCG infants their status may be classified as a ‘contact’ (exposed but not yet tested), ‘latent’ (infected but asymptomatic), or ‘active infection’ (showing symptoms).”or complicated (disseminated) because late at diagnosis or take BCG and already have disease.

There is another critical issue if postpone 6 months that’s mean there is percentage of babies are unprotected with BCG vaccine become close contacts to active tuberculosis patients inside their family like mother or father or relatives or house officer that’s has big hazard on infants health.

## Conclusion

We commend the authors’ contribution to understanding BCG complications in SCID. However, we urge that these data not be used to endorse a 6-month delay as a national or global model.
